# Systems thinking in action: Lessons from Jharkhand's journey to reducing maternal mortality^☆^

**DOI:** 10.1016/j.dialog.2025.100265

**Published:** 2025-12-13

**Authors:** Sanjeev Kumar, Anand Kumar, Amit Kumar Goyal, Ramkrishna Kumar, Pranav Bhushan, Sarwar Khan, Ajit Kumar Singh, Vikas R. Kehsri

**Affiliations:** a Mamta-Health Institute for Mother and Child, New Delhi 110048, India; b JHPIEGO, Bihar, India; c International Institute for Population Sciences, Mumbai 400088, India; d JHPIEGO, Jharkhand, India; e University of Manitoba, Canada; f Azim Premji Foundation, Bihar, India; g National Health System Resource Centre, New Delhi, India; h Jindal School of Public Health and Human Development, O P Jindal Global University, Sonipat, Haryana, India

## 1 Systems thinking in healthcare

Systems thinking is a comprehensive analytical method, historically utilized across diverse areas of computer science, management, and ecology, and is increasingly significant in health policy and systems research (HPSR) [1]. The renewed global focus on systems thinking in health programs is crucial for addressing complex health challenges, such as maternal health [2]. It enables a detailed analysis of health system operations, identifying key relationships and synergies affecting priority health service delivery. It highlights areas of system success and failure, determining integrated strategies for overall health system strengthening [3]. The World Health Organization's health systems framework outlines six interdependent building blocks: service delivery, health workforce, health information systems, access to essential medicines, financing, and leadership and governance [4]. These components must work in tandem, applying a systems thinking approach to address multifaceted challenges. Maternal healthcare is an essential health service that requires the synchronized functioning of all health system's building blocks and the application of systems thinking.

## 2 The context

Jharkhand, a state in eastern India with a population of 39.96 million, of which 26.2 % belong to indigenous communities [5,6], continues to face significant health system challenges. The healthcare delivery structure is organized into three levels. At the primary level, services are provided through Sub-Centres and Primary Health Centres (PHCs), which are intended to deliver preventive, promotive, and basic curative care. At the secondary level, Community Health Centres (CHCs) and District Hospitals (DHs) act as referral units and provide specialist services. At the tertiary level, care is delivered through Government Medical Colleges, which also serve as teaching and training institutions.

Despite this structured framework, the system struggles with severe human resource shortages. As of the Comptroller and Auditor General of India (CAG)audit report, 61 % of specialist and medical officer posts, 52 of staff nurse posts, and 80 % of paramedic posts remained unfilled, undermining service delivery at both PHCs and CHCs [7]. Persistent drug stock-outs further compound these gaps. Between 2019 and 20 and 2021–22, 77 % to 88 % of essential medicines were not procured, leaving facilities unable to meet basic requirements. The Comptroller and Auditor General's audit revealed alarming deficits across different levels of care. In test-checked hospitals, operating theatres stocked only 9–74 % of required drugs, intensive care units had just 5–8 of the 14 essential medicines, and maternity wards lacked critical drugs such as Hydralazine and Methyldopa [7].

**Fig. 1:**
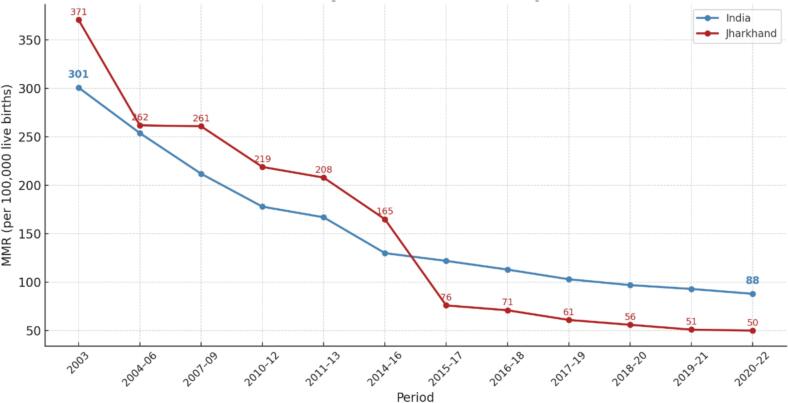
Maternal mortality ration trend-India and Jharkhand.

Despite these systemic limitations, the state has made remarkable progress in reducing the Maternal Mortality Ratio (MMR). Aligned with SDG 3: Ensure healthy lives and promote well-being for all and Target 3.1, Jharkhand reduced its MMR from 371 in 2003 to 50 in 2020–2022 (Fig. 1), achieving an average annual rate of reduction of 11.33 % [8,9]. Therefore, an analysis of MMR trends in Jharkhand, with possible systemic factors, could offer important lessons for other states in India and regions with a similar context. To gain a deeper understanding of the approach and context, a group of collaborators with contextual knowledge of Jharkhand, including implementers, researchers, funders, and community-based institutions, came together to compile the strategies and contextual factors that could have contributed to this progress over time. Drawing on authors' experience and existing literature, we've attempted to explore three key issues regarding the reduction in MMR in Jharkhand.

1. Maternal health Service delivery design and Referral pathway
2. Programs/interventions implemented to improve access, coverage, and quality
3. Application of the Three-Delay Model

## 3 Maternal health service delivery design and referral pathway

The maternal health service organizations in Jharkhand reflect a constant interplay between governance, regulation, and service provision. The Ministry of Health and Family Welfare (MoHFW) at the national level designs and oversees major maternal health programs such as the Janani Suraksha Yojana and the Janani Shishu Suraksha Karyakram, under the National Health Mission. As health is a state subject, the Department of Health, Medical Education, and Family Welfare in Jharkhand is nodal agency for contextualizing and executing these national schemes while mobilizing state resources to strengthen maternity services across communities and facilities. This dual structure of central guidance and state implementation shapes how maternal health programs are delivered and adapted to local needs.

**Fig. 2:**
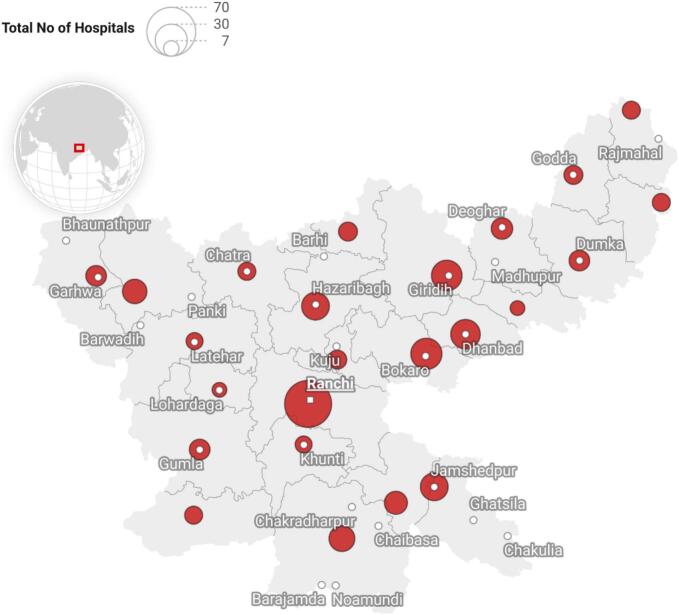
District-wise PMJAY empaneled maternity hospitals.

Within this governance framework, Jharkhand has developed a mixed health system that is more competitive than complementary. While limited partnerships exist—such as contracting private vehicles for the Mamta Vahan referral transport program or outsourcing diagnostic services—maternal care is often characterized by diverting patients from public health hospitals to private hospitals/providers. Families perceive private hospitals and clinics to offer better infrastructure and specialist availability, though affordability remains a barrier. The private providers itself are fragmented and weakly regulated. Although the Clinical Establishments Act (CEA) was adopted in Jharkhand in 2013, its enforcement has been minimal, often limited to provisional registration managed by district Chief Medical Officers. Even after the introduction of Ayushman Bharat – Pradhan Mantri Jan Arogya Yojana (PMJAY), which empanelled both public and private facilities for maternity care with insurance coverage of up to ₹5 lakh per household, effective implementation of CEA provisions remains weak(Refer to Fig. 2). As a result, competition rather than complementarity continues to define the interaction between public and private providers in maternal healthcare.

### 3.1 Referral pathway

**Fig. 3:**
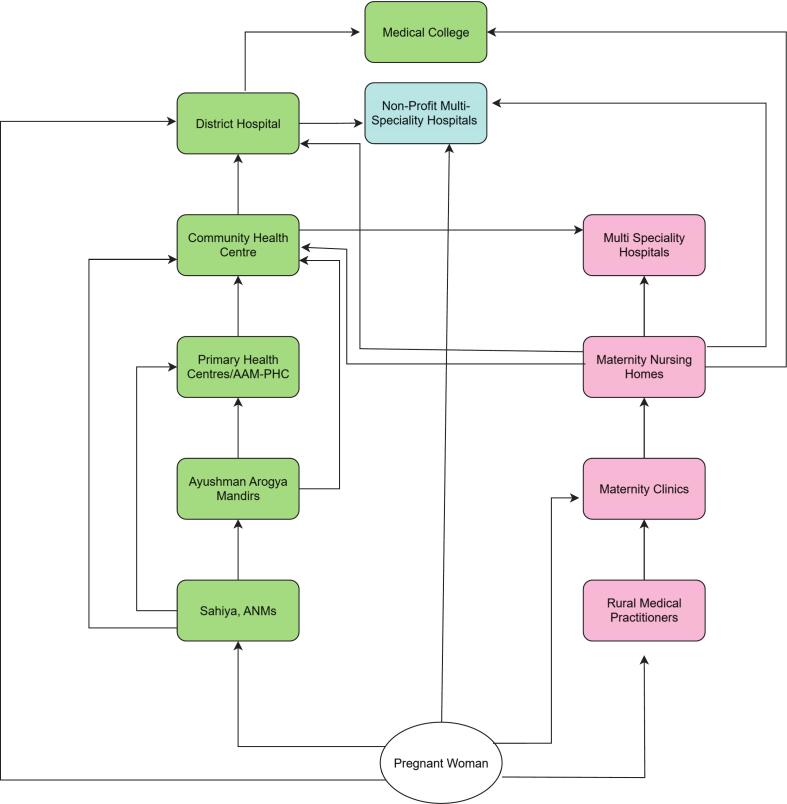
Referral pathway.

Maternal healthcare delivery in Jharkhand is organized through a three-tiered system designed to ensure continuity of care from community to tertiary care facilities. At the community level, Sahiyas (ASHAs), Auxiliary Nurse Midwives (ANMs), and Sub-Centres form the backbone of the public health system. It offers antenatal care registration, health education, and referral assistance. In remote and underserved areas, informal providers such as Registered Medical Practitioners (RMPs) and dais often act as the first point of contact, referring women onward to formal facilities (Refer to Fig. 3).

At the primary level, Primary Health Centres (PHCs) and small private clinics provide routine obstetric services and manage uncomplicated deliveries. Emergency/critical cases are referred to Community Health Centres (CHCs) and Sub-Divisional Hospitals, which serve as the secondary level and are equipped to provide basic and some comprehensive emergency obstetric care. Private nursing homes also operate at this level and offer basic and CEmoC services.

At the tertiary level, district Hospitals and medical colleges act as referral centres for the district, with obstetric high-dependency units, blood banks, and intensive care services. Private multi-specialty hospitals provide obstetric emergency services in parallel.

This layered public and private maternal health system, complemented by referral transport schemes such as the National Ambulance Service and Mamta Vahan, forms the backbone of Jharkhand's maternal health referral pathway.

## 4 Programs/interventions implemented to improve access, coverage, and quality for maternity care services

With the launch of the Reproductive and Child Health Phase 2 on April 1, 2005, and the National Rural Health Mission launch the same year, the state has recorded significant improvements in reducing maternal mortality. Several Programs and interventions across various components of Maternal Health are being implemented to ensure comprehensive coverage and effectiveness across facilities of public, private, and Non-Governmental Organizations. Potentially, these programs/ interventions have contributed to this reduction in maternal mortality.

To further describe the roles of these programs, we identified 21 interventions implemented since 2004 across public, private, and non-governmental organizations, based on intermediate health system outcomes such as access, coverage, and quality (see Table 1).

**Table 1:** Programs/ interventions implemented in Jharkhand.

Sr. No.	Target intermediate outcome (health system)	Sector (public/private)	Program name	Objective	Intervention details	Launched in	Implementation status
1	Access	Public	Janani Suraksha Yojana (JSY)	Promote institutional deliveries among pregnant women to reduce maternal and neonatal mortality	Conditional cash transfer to pregnant women for delivering in public health facilities, with mobilization by Sahiya's [10]	2005	Ongoing
2	Access	Public	Mobile Medical Units	To improve the health services at the village level for the most underserved communities living in difficult-to-reach areas.	Provide primary care services for common diseases, including communicable and non-communicable diseases, and RCH services; carry out screening activities and refer to appropriate higher faculties [[10], [11], [12]]	2008	Ongoing
3	Access	Public	Janani Shishu Suraksha Karyakram (JSSK)	Ensure free and non-expense delivery (including C-section) and newborn care in public health institutions	Free drugs, consumables, diagnostics, diet, blood, and transportation (home-institution, inter-facility, drop-back) for pregnant women and sick newborns up to 1 year old [10].	2011	Ongoing, universal in the public sector
4	Access	NGO	Oxfam-DFID & CINI Integrated Maternal Health	Build community capacity to demand, access, and monitor maternal health services	Community mobilization, capacity building, nutrition, maternal health education, and monitoring of public services in partnership with CINI (Child in Need Institute) [13]	2012	Completed, model replicated
5	Access	Public	Obstetric High-Dependency Units & Intensive Care Units	To improve access to emergency obstetric care at district hospitals and medical Colleges	Establishment of Obstetric High-Dependency Units in all district hospitals and Obstetric ICUs in all medical colleges [14].	2016	Ongoing
6	Access	Public	Operationalizing First Referral Units	To strengthen facility-based emergency obstetric care. The facility includes Community Health Centres, Sub-divisional Hospitals, and district hospitals.	Minimum services to be provided by a fully functional FRU [15]: A. 24-h delivery services, including normal and assisted deliveries B. Emergency Obstetric Care, including surgical interventions like Caesarean Sections and other medical interventions C. Newborn Care D. Emergency Care of Sick Children E. Full range of family planning services, including Laparoscopic Services F. Safe Abortion Services G. Treatment of STI / RTIH. Blood Storage Facility I. Essential Laboratory ServicesJ. Referral (transport) Services	2004	Ongoing
7	Access	Public	Mamta-Vahan	To ensure a referral vehicle for pregnant women for institutional delivery	At the panchayat level, private vehicle owners enter agreements with the government, thus expanding the fleet of vehicles available for the scheme. Under this scheme, pick-up and drop-off services are provided for pregnant women [16].	2011	Ongoing
8	Access	Public	National Ambulance Service	To establish a responsive and accessible emergency medical transportation system	It offers emergency and patient transport via the 108 and 102 ambulance services. Dial 108 handles urgent responses for critical care, trauma, and accidents, whereas Dial 102 primarily caters to pregnant women, children, and other vulnerable groups. The service operates around the clock, with ambulances equipped with GPS and life-saving medical instruments and equipment [17].	2012	Ongoing
9	Access, Community Mobilization	Public	Sahiyya	To mobilize communities for accessing health services, including maternal health	Participate in developing village health Plan; Communication for health behavior change; Counselling, including escorting patients to the hospital, providing primary medical care for identified conditions; Act as depot holders, maintaining records and maintaining register for every birth, death, daily work, etc. [18].	2005	Ongoing, state-wide
10	Access, Coverage	NGO/CSR	Maternal and Newborn Survival Initiative (MANSI)	Reduce neonatal and maternal mortality through community-based interventions	Home-based newborn and maternal care, community health worker training, partnerships with Tata Steel, the American India Foundation, and the local health system, focusing on rural blocks [19,20].	2011	Ongoing, expanded to multiple districts
11	Access, Coverage, Quality	NGO	PPIA by Transform Rural India (TRI)	Bridge the policy-to-practice gap, improve rural maternal health	Young professionals (PPIA fellows) deployed in rural areas to support local governments, set up ANC Corners in Anganwadi Centres, and develop tailored solutions for maternal health challenges [21,22].	2022	Ongoing, West Singhbhum and beyond
12	Access, Quality	Private/NGO	Health Voucher Scheme (Chief Minister Janani-Shishu Abhiyaan)	Increase access and choice for poor women; incentivize institutional delivery and maternal care	Demand-side financing: vouchers for early registration, institutional delivery, immunization, and provider facilitation; vouchers can be redeemed at empaneled private or public providers; aims to increase private sector presence and induce quality competition [23].	2011	Piloted
13	Coverage	Public	Pradhan Mantri Matru Vandana Yojana (PMMVY)	Provide partial wage compensation and improve the nutritional status of pregnant and lactating women	Cash incentives for first live birth, conditional on early registration, ANC, and child immunization [10,24].	2017	Ongoing, national coverage
14	Coverage	Public	Pradhan Mantri Surakshit Matritva Abhiyan (PMSMA)	Provide assured, comprehensive, quality antenatal care to all pregnant women in the 2nd & 3rd trimesters	Fixed-day (9th of every month) ANC clinics at public facilities, specialist check-ups, essential investigations, early detection of high-risk pregnancies; Extended PMSMA (e-PMSMA) for additional follow-up visits and high-risk pregnancy tracking, with fixed-day service on the 21st of every month [10,25].	2016 (e-PMSMA 2022)	Ongoing, expanded for HRP tracking
15	Coverage; Quality	Public	Skilled Birth Attendant Training	To upskill healthcare providers with the necessary skills to manage normal pregnancies, childbirth, and immediate postpartum periods, and identify and manage complications.	This is a structured, skill-based initiative designed to enhance the competencies of healthcare providers, including Auxiliary Nurse Midwives (ANMs), Lady Health Visitors (LHVs), and Staff Nurses (SNs), in delivering quality antenatal, intrapartum, and newborn care, with a focus on safe delivery and the management of complications [26,27].	2008	Ongoing
16	Quality	Private	Manyata	Improve the quality and safety of maternity care in private facilities	The voluntary certification program for private maternity facilities includes nurse training on lifesaving practices, mentoring support, and assessment and certification for meeting quality standards. Implemented by Jhpiego, supported by MSD for Mothers and FOGSI [28].	2016	Ongoing, voluntary participation
17	Quality	Public	Maternal Death and Surveillance and Response	To strengthen the mechanisms and processes for Maternal Death Surveillance & Response	MDSR is a continuous cycle of identification, notification, and review of maternal deaths, followed by actions to improve the quality of care and prevent future deaths [29].	2017	Ongoing
18	Quality	Public	LaQshya (Labour Room Quality Improvement Initiative)	Improve the quality of care in labour rooms and maternity OTs for respectful and safe childbirth	Infrastructure upgrades, essential equipment, staff capacity building, adherence to clinical guidelines, and quality assurance processes in labour rooms and OTs [10,30].	2017	Ongoing, implemented in key facilities
19	Quality	Public	SUMAN (Surakshit Matritva Aashwasan)	Assured, dignified, respectful, and quality healthcare at no cost for every woman and newborn	Zero tolerance for denial of services; focus on respectful care, prompt emergency response, and accountability [10,31].	2019	Ongoing, phased implementation
20	Quality	Public	Dakshta	To strengthen the competency of providers of the labour room, including medical officers, staff nurses, and ANMs, to perform evidence-based practices per established labour room protocols and standards.	A three-day training program for clinical update and skills standardization, designed for medical officers, staff nurses, and ANMs, to ensure they perform evidence-based practices by established labour room protocols and standards [32].	2015	Ongoing
21	Quality	Public	Midwifery Services	To provide access to quality maternal and newborn health services and promote natural birthing by promoting a positive childbirth experience	Midwifery-led Care Units are being set up at high-volume, ‘LaQshya’-certified public health facilities. Midwifery program trainees will be posted here to offer 24/7 delivery services. Midwives must be assigned as a team, not individually, since the impact of the midwifery program on care quality can only be measured if Midwifery-led Care Units are operational [33].	2018	Ongoing

**Access:** Improving access to maternity care has been a priority in the public health system, with several large-scale programs implemented. The operationalization of First Referral Units (FRUs) and the setting up of Obstetric High Dependency Units at district hospitals have brought emergency services closer to rural and underserved populations. Transportation barriers have been addressed through the National Ambulance Service (Dial 108/102) and the Mamta-Vahan initiative. Mamta Vahan is a decentralized referral system that uses private vehicle owners at the panchayat level. Financial access has been enhanced by schemes like Janani Shishu Suraksha Karyakram (JSSK) and Janani Suraksha Yojana (JSY), which provide cash incentives and free care—including C-sections, diagnostics, and transportation—for pregnant women and newborns. NGOs and CSR partners also play a crucial role, as exemplified by MANSI (Tata Steel-AIF) and Oxfam-CINI, which focus on community mobilization and support in remote, tribal areas. The Health Voucher Scheme, led by the private/NGO sector, helps poor women access healthcare through redeemable service vouchers at empaneled facilities. It facilitates access to maternity services with choice and competition, on a limited scale.

**Coverage**: Both government and non-government organizations have introduced several initiatives to improve maternal health coverage. The Pradhan Mantri Matru Vandana Yojana (PMMVY) provides partial wage support to pregnant women and encourages early antenatal registration. The Pradhan Mantri Surakshit Matritva Abhiyan (PMSMA) offers specialist-led antenatal check-ups on fixed days, with follow-up tracked through the e-PMSMA system. To reach women in hard-to-access areas, Mobile Medical Units deliver reproductive, child health, and non-communicable disease services. NGOs are also playing an important role — for example, MANSI and Transform Rural India's PPIA deploy community health workers and medical professionals to extend outreach in rural Jharkhand, set up antenatal care corners at Anganwadi Centers, and maintain continuity of care. These combined public–NGO efforts have helped bring services closer to the most underserved women, including those who are high-risk, marginalized, or living in remote tribal regions.

**Quality:** The drive for quality in maternal care has led to the implementation of comprehensive training, accreditation, and mentoring programs, mainly led by the public sector but increasingly supported by private and non-government organizations. Programs such as Skilled Birth Attendant Training, Dakshta, and LaQshya focus on skill-building and adherence to clinical protocols in labor rooms. The Maternal Death Surveillance and Response (MDSR) system ensures learning from every maternal death to prevent future cases. LaQshya-certified public hospitals promote positive childbirth experiences and are staffed by specially trained professionals who provide round-the-clock services. The Manyata initiative, a voluntary certification for private facilities supported by Jhpiego and Federation of Obstetric and Gynecological Societies of India (FOGSI), ensures quality in the private maternity facilities. Meanwhile, the SUMAN program (Surakshit Matritva Aashwasan) institutionalizes dignified, respectful, and zero-denial care across all public health facilities.

## 5 Application of the three-delay model in the context of Jharkhand

To better understand how interventions align with the Three Delay Model in reducing maternal mortality, we used the framework as a guide and examined Jharkhand's efforts, showing how they correspond to its principles.

### 5.1 Application of the three delay model

This globally recognized framework highlights three critical points at which delays can occur, each of which can significantly increase the risk to a mother's life during pregnancy and childbirth [15].

The first delay happens when there is hesitation or failure to seek care, often because of limited awareness or an inability to recognize danger signs during pregnancy. Long-standing sociocultural norms frequently shape decisions. In Jharkhand, this is being addressed through community-based initiatives led by Sahiyas. Through local awareness programs such as Village Health Sanitation and Nutrition Committees (VHSNCs) and targeted behavior change communication (BCC) and information, education, and communication (IEC) activities, Sahiyas are helping women and families identify complications early and make timely decisions to seek care (see Table 2).

**Table 2:** Summary-interventions categorization based on 3-delay models.

Delay	Description	Key Barriers	Jharkhand Interventions
Delay 1(Delay in deciding to seek care)	Delay occurs at the household or community level when a woman or her family fails to recognize the need for care or decides to seek help too late.	Lack of awareness of danger signs- Sociocultural norms- Low status of women- Limited decision-making power	Sahiyya and its Movement- Awareness campaigns (VHSNCs, BCC/IEC)- Community mobilization and engagement
Delay 2(Delay in reaching a healthcare facility)	Delay in physically accessing a health facility after the decision to seek care has been made.	Distance to health facilities- Lack of transportation- High costs- Poor referral linkages	Health voucher schemes
			Strengthened referral systems and linkages through Free ambulance services (National Ambulance Service, Mamta Vahan)
Delay 3(Delay in receiving adequate care at the facility)	Delays occur within the health facility when timely and quality care is not provided.	Poor quality of care- Shortage of skilled staff- Inadequate supplies and infrastructure- Disrespectful or negligent care	LaQshya initiative- SUMAN program
			Manyata certification (private sector)
			Strengthening Emergency Obstetric Care (EmOC) at FRUs,
			Establishment of obstetric High dependency units and Obstetric ICUs in Medical Colleges

The second delay occurs in reaching an appropriate healthcare facility. Factors such as poor transportation, long distances, high costs, and the absence of ambulances often contribute to this delay. Jharkhand has implemented programs/interventions to overcome these factors, like the National Ambulance Service and Mamta Vahan. These provide free transportation, alongside health voucher schemes that alleviate financial burdens. Strengthened referral systems also ensure women can access higher-level care quickly when needed.

The third delay occurs within the health facility, where women may not receive timely or adequate care due to a shortage of skilled staff, inadequate supplies, and insufficient infrastructure. To address this, Jharkhand introduced facility-level programs such as LaQshya and SUMAN and promoted Manyata certification in private hospitals. These measures focus on improving emergency obstetric care by strengthening First Referral Units, MCH wings, high-dependency units at district hospitals, and obstetric ICUs in medical colleges. LaQshya also emphasizes continuous quality improvement to ensure care is both safe and respectful.

Through these combined efforts of public institutions, private providers, and NGOs, Jharkhand has potentially developed a system-wide approach for improving maternity care services. By addressing each delay, Jharkhand demonstrates how integrated strategies can save lives and improve maternal health outcomes.

## 6 Transferability and generalizability: lessons for underserved populations

Jharkhand's experience offers insights for other resource-constrained settings, particularly those serving underserved and marginalized populations. The state's context—marked by a high proportion of indigenous communities (26.2 %), persistent shortages of human resources for health (with 61 % of specialist posts and 80 % of paramedic posts vacant), frequent stock-outs of essential medicines (77–88 %), and longstanding governance challenges—mirrors conditions found in many low-resource regions across India and globally. The interventions adopted in Jharkhand were not resource-intensive and relied primarily on community-based, decentralized mechanisms. Community mobilization through Sahiyas supported by structured supervision and incentives, the decentralized Mamta Vahan referral transport system using locally available private vehicles, and participatory community learning and action approaches collectively demonstrated effectiveness under constrained conditions. Notably, the participatory learning and action model, when implemented across 20 districts, achieved a 24 % reduction in neonatal mortality and remained cost-effective, underscoring the feasibility of scaling community-driven strategies in low-resource environments.

Despite this transferability, several context-specific considerations merit attention when adapting Jharkhand's model to other settings. Sustained political commitment and targeted financing were central to the state's progress; replication in fiscally weaker regions requires a realistic appraisal of local financing capacity and political will. Moreover, Jharkhand itself is heterogeneous; variations in outcomes across tribal and non-tribal areas were significant but not fully explored in this synthesis, limiting clarity on which interventions were most effective for specific subgroups. The role and density of private providers also vary widely across regions, influencing how public–private interactions and regulatory mechanisms may be adapted.

For these reasons, policymakers seeking to apply Jharkhand's lessons should undertake rapid context assessments to identify alignment with local governance arrangements, fiscal constraints, demographic characteristics, and health system capacity. Implementation strategies may then be sequenced and calibrated based on local absorptive capacity. Framed in this way, Jharkhand's experience serves as a flexible, pragmatic guide rather than a prescriptive blueprint, offering actionable pathways to improve maternal health outcomes in other underserved, high-burden settings.

## 7 Strengths and limitations

This commentary highlights how Jharkhand strengthened maternal care in a stepwise manner—from community outreach by Sahiyas to dependable transport through Mamta Vahan and upgraded services under FRUs, LaQshya, and SUMAN—making care more accessible and trustworthy for women. Applying the Three-Delay lens shows how each intervention helps reduce risk and offers practical lessons for other settings facing staff shortages, stock-outs, and weak regulatory environments.

At the same time, the commentary has some limitations. It mainly draws on the author's experience and secondary sources rather than on district- or facility-level data that link program coverage or quality improvements to changes in mortality. The use of the Three-Delay model is descriptive and lacks delay-specific indicators, such as referral completion, travel time, or in-facility response times, leaving some pathways unverified. The discussion of private-sector roles is also qualitative, with limited data on service volumes, outcomes, or quality, which makes their contribution to overall system performance less specific.

**Future Research:** This commentary is designed as a narrative piece. It aims to draw practical lessons from Jharkhand's success story, not to measure program impacts. Going forward, the evidence base for different program impacts can be strengthened through future studies such as:

1. Longitudinal cohort studies that track knowledge and skill retention among community health workers and facility staff at baseline, 6, 12, and 24 months after training. These studies can also assess on-the-job performance and quality of care.
2. Prospective facility audits that examine adherence to LaQshya, SUMAN, and MDSR standards over 3–5 years. Audit findings can be linked to maternal and neonatal mortality trends at the facility level.
3. Community behavioral cohort studies that follow women and families in intervention and comparison areas. These can measure awareness, care-seeking practices, and decision-making delays through repeated data collection during pregnancy and the postpartum period.
4. Equity-focused impact assessments conducted prospectively across tribes, castes, wealth groups, and geographic zones. These can help identify differences in knowledge retention and program reach.

## 8 Conclusion and way forward

Efforts to improve maternity services and reduce maternal deaths in the state have shown notable results. A key factor has been the focused strengthening of Sahiyas through regular on-the-job training, expanded roles, and better planning of their service areas. These steps have helped Sahiyas mobilize communities, run women's group meetings, and share important maternal health messages more effectively. Ongoing supportive supervision and performance-based incentives have also helped keep frontline workers motivated and effective, even in remote tribal areas.

At the same time, the Mamta Vahan scheme — a government-run ambulance and referral transport service — has been vital in improving outcomes for mothers and newborns. By tackling the “second delay” in the three-delays model —the challenge of reaching proper care in time —the scheme has made it easier for pregnant women to reach health facilities quickly. This has increased access to safe, facility-based deliveries, especially for women in rural and marginalized communities, and has been widely recognized as an essential factor in reducing maternal deaths by ensuring timely care for obstetric emergencies.

Evidence from the participatory learning and action (PLA) approach highlights the value of community-led strategies. Rolled out in 20 of the state's 24 districts, PLA was linked to a 24 % drop in neonatal deaths and proved highly cost-effective, demonstrating how participatory methods can make a real impact on maternal and newborn health at scale.

However, challenges remain. While maternal deaths have decreased, progress is uneven, with apparent differences between regions and social or economic groups. The quality of care in health facilities varies widely, and weak referral and communication systems often delay timely access to care. Without addressing these systemic weaknesses, further reductions in maternal mortality could be limited.

Addressing these challenges will require targeted, multi-pronged strategies. Expansion of mobile health units and outreach services for underserved tribal populations may help reduce geographic inequities in access to maternity services. The quality of facility-based care can be improved through regular audits, supportive supervision, continuous professional development, and simulation-based training, all of which are reinforced by accountability mechanisms and patient feedback systems.

## CRediT authorship contribution statement

**Sanjeev Kumar:** Writing – review & editing, Writing – original draft, Visualization, Methodology, Investigation, Formal analysis, Data curation, Conceptualization. **Anand Kumar:** Writing – original draft, Formal analysis, Data curation. **Amit Kumar Goyal:** Writing – original draft, Visualization, Data curation. **Ramkrishna Kumar:** Writing – original draft, Visualization, Data curation. **Pranav Bhushan:** Writing – original draft, Formal analysis, Conceptualization. **Sarwar Khan:** Writing – review & editing, Validation. **Ajit Kumar Singh:** Writing – review & editing. **Vikas R. Kehsri:** Writing – review & editing, Supervision.

## Declaration of competing interest

The authors declare that they have no known competing financial interests or personal relationships that could have appeared to influence the work reported in this paper.

The author is an Editorial Board Member/Editor-in-Chief/Associate Editor/Guest Editor for this journal and was not involved in the editorial review or the decision to publish this article.
